# Bedaquiline (BDQ) resistance in an adolescent with multidrug-resistant tuberculosis (MDR-TB): An alarm for pediatricians

**DOI:** 10.1016/j.idcr.2023.e01880

**Published:** 2023-08-29

**Authors:** Citra Cesilia, Muh Akbar Tirtosudiro, Heda Melinda Nataprawira

**Affiliations:** Division of Respirology, Department of Child Health, Faculty of Medicine, Universitas Padjadjaran, Indonesia

**Keywords:** Bedaquiline resistance, Children, Indonesia, Multidrug resistance, Tuberculosis

## Abstract

Bedaquiline (BDQ) use for all age groups in drug-resistant (DR) tuberculosis (TB) regimens for children may be shorter, safer, and more effective. However, the emergence of BDQ resistance reports soon after its introduction is alarming. We report the case of a 17-year-old boy, initially diagnosed with Rifampicin-resistant (RR)-TB and developed BDQ resistance during the treatment. To the best of our knowledge, this is the first report of BDQ resistance in pediatric

## Introduction

According to the World Health Organization (WHO) data for 2021, tuberculosis (TB) remains the primary cause of death among infectious diseases globally. Each year, the estimated number of TB cases worldwide exceeds 10.6 million, marking a 4.5% increase compared to the 10.1 million cases in 2020. TB can affect individuals of all age groups. In 2021, the prevalence of TB in children was reported to be 11%, with mortality rates of 11% for those who were HIV-positive and 14% for those who were HIV-negative [1].

Prevalence of drug-resistant (DR)-TB in children is unknown. Estimated incidence by WHO had reported an increase of DR-TB cases worldwide between 2020 and 2021 in comparison to between 2015 and 2020 when the estimated annual number of individuals globally diagnosed with multidrug-resistant (MDR)-TB or Rifampicin-resistant (RR)-TB remained relatively constant. However, there was a 3.1% growth in 2021 compared to 2020. In 2010, approximately 32,000 children under 15 were diagnosed with DR-TB worldwide. It is estimated that the majority of these cases, around 95%, remain undiagnosed due to a phenomenon known as the notification gap. The breakdown of tuberculosis types is as follows: 13.59% for RR-TB, 3.72% for MDR-TB, 6.07% for monoresistant (MR)-TB, 1.61% for polyresistant (PR)-TB, and 0.44% for extensively drug-resistant (XDR)-TB [2].

DR-TB has become a challenge and essential problem in managing paediatric TB, even before the COVID-19 pandemic. The Coronavirus Disease (COVID)− 19 pandemic has severely impacted the detection and reporting of TB cases, especially in children. Low case detection will impact late diagnosis and treatment, which can increase the morbidity and mortality of TB in children, particularly DR-TB [3]. So far, the estimation of the incidence of TB in children is generally an epidemiological model that is extrapolated from the available data from the number of TB cases or DR-TB of adult patients. In countries with low resources, disruption to access to healthcare facilities, limited facilities, and lack of public awareness and/or education about TB can cause different numbers to be reported or underreporting. Regarding the facilities that are limited in number and quality, methods and protocols for the detection of close contacts in patients with a previous history of TB is still relatively scarce, which can result in the number of DR-TB diagnoses that is likely to be lower than it is [3], [4], [5]. In addition to the low case detection rate and high mortality rate, difficulties in sampling, paucibacillary characteristics in children, and therapy that is not child-friendly are the biggest challenges in treating DR-TB children worldwide [2].

Bedaquiline (BDQ) is an antimicrobial with novel mechanisms of action against *Mycobacterium tuberculosis*. Promising results from DR-TB regimens including BDQ had reported shorter, less toxic, and more effective compared to conventional DR-TB regimens [6]. In 2012, the United States Food and Drug Administration (US FDA) Centre for Drug Evaluation and Research (CDER) had approved the drug for treatments of DR-TB in adults. In 2013, WHO had issued a recommendation for its use in treating patients with MDR-TB [7] .Since March 2022, BDQ had been recommended by the WHO for use in all age groups [6]. Previously, the use of BDQ was restricted to children below the age of six years due to a lack of comprehensive studies on its effectiveness in older children [8]. Conversely, reports of BDQ resistance in adults have been abundant [9], [10], [11]. In this report, we present the case of a 17-year-old boy initially diagnosed with RR-TB but later found resistance to BDQ based on drug-susceptibility test (DST). To the authors' knowledge, this is the first case report of BDQ resistance in children in Indonesia.

## Case report

A 17-year-old boy has referred from the community health services with chief complaints of haemoptysis one month ago. The complaint was preceded by a persistent cough four months ago, fever for more than two weeks, night sweats, occasional chest pain, and unintentional weight loss of 5 kg in a month. No contacts were identified, and he had never been diagnosed with TB.

**Fig. 1:**
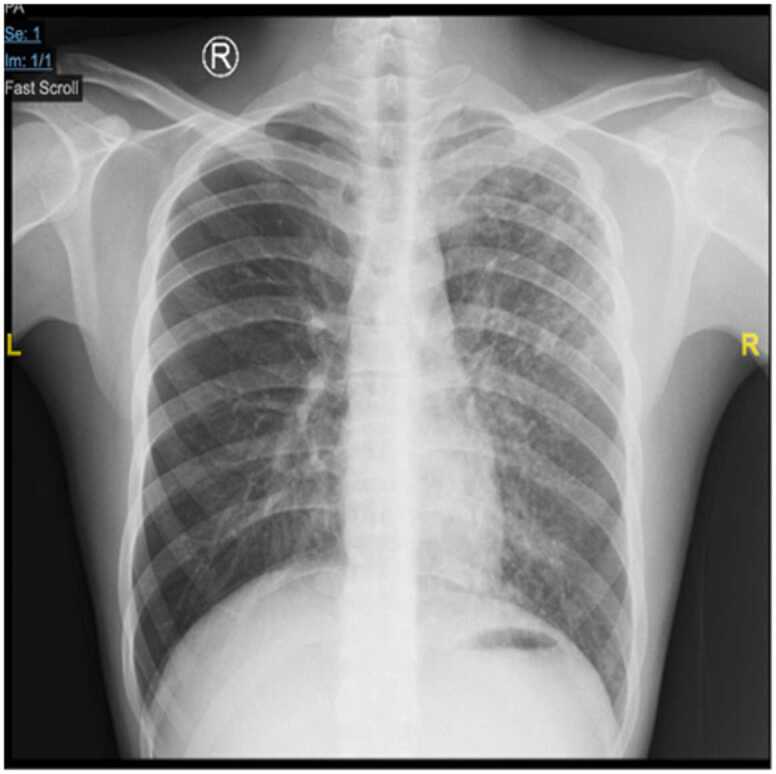
Chest X-ray on admission. Infiltrates were seen in the field above to below the left lung.

On physical examination, he appeared mildly ill. Nutritional status and vital signs were within normal limits. Anti-HIV examination showed negative results, while other baseline examinations before administration of the MDR-TB regimen were within normal limits. The chest x-ray (CXR) shows an infiltrate in the middle to lower left lung field without any cavity (Fig. 1). The acid-fast bacilli (AFB) test results were negative, while the GeneXpert MTB/Rif results consistently showed low detected MTB with Rifampicin resistance was detected. The line-probe assay (LPA) results showed that fluoroquinolone and second-line injectable drugs were effective. He was diagnosed with MDR-TB and given a shorter regimen of all drugs from Group A (Levofloxacin, Linezolid, and Bedaquiline) and B (Clofazimine and Cycloserine) for 9 – 11 months.

**Fig. 2:**
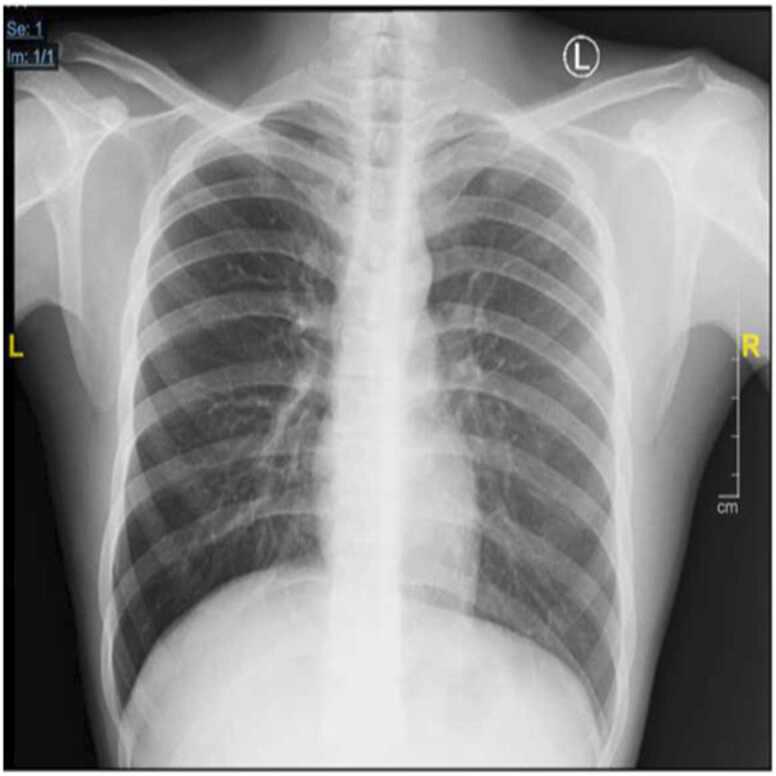
Chest X-ray on 4th month of treatment. Infiltrates below the left lung decreased.

In the fourth month of treatment, the DST had detected resistance to BDQ and high dose Isoniazid (HHD); no resistance to levofloxacin (Lfx), high dose Moxifloxacin (Mfx HD), Linezolid (Lzd), Amikacin (Amk), Clofazimine (Cfz), and Pyrazinamide (Z) were detected. The treatment was declared as a failure; the treatment was substituted into a longer-term regimen consisting of Lfx, Lzd, Cfz, cycloserine (Cs), and Z for 18 – 24 months. Whole genomic sequencing (WGS) was also performed; the sample was excluded at the screening due to negative sputum culture results. During monitoring, the patient experienced clinical improvement from CXR (Fig. 2), culture examination underwent conversion, and the patient was planned for 18 months of treatment.

## Discussion

Our patient presented with haemoptysis for one month, preceded by a persistent cough for four months. Other symptoms indicative of TB such as low-grade fever and weight loss were reported. Haemoptysis may present as a severe complication of pulmonary tuberculosis (PTB), whether treated or left untreated [12], [13]. Haemoptysis can be caused by various factors, including bleeding from the cavity wall, endobronchial tuberculosis (EBTB), post-TB bronchiectasis, aspergilloma, or the rupture of Rasmussen's aneurysm [13], [14]. Bronchial artery involvement in PTB is a frequent underlying cause. Most adolescents exhibit intrathoracic TB, which resembles the adult form of TB [12], [13]. Typical symptoms encompass coughing, haemoptysis, fever, and weight loss. Chest radiographs reveal age-related variations in the pathogenesis: cavitation and pleural effusions are more prevalent in adolescents, while intra-thoracic lymphadenopathy or miliary disease, as observed in younger children, are less frequent. Moreover, parenchymal changes in adolescents are more prone to appear apically, resembling those in adults [15], [16] This is along with CXR findings which revealed infiltration in hemithorax bilaterally and intra-thoracic lymphadenopathy.

There are two ways in which a child can develop MDR-TB: primary or transmitted resistance and acquired resistance. Most children with MDR-TB have primary resistance, with only a few young children acquiring resistance [17]. The patient had no history of TB and we could not identify any TB close contacts within the patient's family. Contact investigations are difficult to carry out due to limited examination modalities for contact investigations in primary health facilities [12]. Based on Indonesian Contact Investigation Guidelines, contact investigation should be performed by a minimum of 20 persons around the case index [18].

Nutritional status of the patient was normal with negative HIV status. The CXR results did not find extensive lung damage. Considering the demonstrated efficacy of fluoroquinolones in the second-line LPA findings, we selected a brief treatment regimen comprising five medications from Group A (Lfx, Lzd, and Bdq) and all the medications from Group B (Cfz and Cs) [8], [14], [19].

The phenotypic culture in our hospital utilizes solid media, resulting in a prolonged turnaround time of approximately 8–12 weeks for obtaining results. In the fourth month of treatment, the culture drug sensitivity test (DST) results showed resistance to BDQ and high doses of isoniazid. Nevertheless, they exhibited susceptibility to fluoroquinolones, Lzd, Cfz, Cs, and Pza. We did not perform a whole genomic sequence (WGS) because this test is not available in our health facilities, so we cannot confirm whether there is resistance to other drugs or cross-resistance to clofazimine. As a result, we categorized the patient as having a treatment failure of MDR-TB. According to the DST findings, we substituted BDQ with pyrazinamide and devised a long-term regimen of a minimum duration of 18 months.

BDQ is crucial as a primary medication in managing RR-TB. Starting from 2012, the importance of BDQ has been growing significantly in treating RR-TB [19], However, the limited availability of standardized BDQ DST worldwide poses a challenge to its efficient use in routine care [20], [21]. Regarding DR-TB management, there are recommendations for using BDQ and delamanid in children, applicable across all age groups [6]. This recommendation updates the WHO 2014 recommendation concerning the use of BDQ in children aged six years and older, while delamanid was recommended for children aged three years and older [8]. Regrettably, reports of BDQ resistance had already emerged shortly after its introduction coupled with challenges in assessing BDQ resistance [9], [10], [11]. The current gold standard using phenotypic DST to assess drug resistance requires up to six weeks to complete while the critical concentration cut-offs are still determined based on interim recommendations instead of standardized normal values [20], [22]. There is no readily accessible, rapid genotypic DST for BDQ, primarily due to the lack of studies regarding possible genetic variants to development of BDQ resistance and its resulting phenotype. Prior catalogued mutations in the MTB complex by WHO in 2021 does not include any genotypic variants potentially associated with BDQ resistance [19].

Current WHO guidelines 2022 recommend prioritizing the standardized shorter all-oral containing bedaquiline regimen for children with MDR/RR-TB. Children who are not eligible for an all-oral bedaquiline-containing regimen should be treated with an individualized longer TB regimen (LTR) consisting of at least four medicines the organism is likely susceptible to [6]. Group A and B medicines should be prioritized in the construction of the treatment regimen, and other Group C medicines. The regimen for susceptible fluoroquinolones is BDQ-Lfx-Lzd-Cfz-Cs. Delamanid, PAS, ethionamide, ethambutol, and pyrazinamide are additional medicines [6]. In this patient, the results of the DST examination showed resistance to Bdq, so we added one drug from group c. Delamanid was not chosen because the availability of the drug was limited, and priority was given to DR-TB patients who had previously received delamanid. Even though PAS showed effectiveness only in regimens without BDQ, Lzd, Cfz or delamanid, it is proposed only when other options to compose a regimen are not possible. We chose pyrazinamide as an additional medicine because there is evidence of susceptibility from DST.

Referring to the technical guidelines for the management of DR-TB in Indonesia in 2020, if drug intolerance occurs in short-term combinations that require discontinuation of one of the primary drugs (Bdq, Lfx/ Mfx, Cfz, Eto, HD-H), then the short-term regimen must be stopped and recorded as failure-to-treat. The patient was subsequently transferred to longer-term treatment based on the following conditions: Firstly, if the patient has achieved culture conversion, the duration of longer-term treatment can be extended by counting the months of treatment completed (e.g., if the patient has undergone three months of treatment and conversion occurs in the second month, treatment should be continued until a total duration of 18 months is reached). Secondly, early initiation of long-term treatment is necessary if the patient requires culture conversion [23].

We emphasize the importance of tailoring regimens for each individual, and regular clinical and laboratory monitoring, to minimize the likelihood of developing BDQ resistance. Furthermore, the availability of genotypic and phenotypic DST will be essential in the treatment of MDR-TB, helping to prevent the emergence of resistance to other antituberculosis medications.

## Ethical approval

Ethical clearance following the Declaration of Helsinki.

## Consent

Written informed consent was obtained from the patientfor publication of this case report and accompanying images. A copy of the written consent isavailable for review by the Editor-in-Chief ofthis journal on request.

## Funding

We thank to 10.13039/501100015690Universitas Padjadjaran for the financial help granted in publishing this article.

## CRediT authorship contribution statement

C.C. drafted the manuscript under the supervision of H.M.N. C.C. and M.A.T contributed to data collection and synchronized. All authors critically revised the manuscript for important intellectual content and approved the final version of the manuscript before submission for publication.

## Declaration of Competing Interest

None declared.
